# Photosensitive symmetric papules of the nasal alae

**DOI:** 10.1016/j.jdcr.2026.01.017

**Published:** 2026-01-21

**Authors:** Dorothy S. Peng, Elaine Dong, Justine Seivright, Gregory Gates, Michael O. Nguyen

**Affiliations:** a David Geffen School of Medicine at University of California Los Angeles, Los Angeles, California; b Division of Dermatology, Department of Medicine, David Geffen School of Medicine at University of California Los Angeles, Los Angeles, California; c Division of Dermatopathology, Department of Pathology and Laboratory Medicine, David Geffen School of Medicine at University of California Los Angeles, Los Angeles, California

**Keywords:** cutaneous lupus erythematosus, dermal mucin, dermatopathology, discoid lupus erythematosus, follicular plugging, interface dermatitis, nasal papules

## Case description

**Fig 1:**
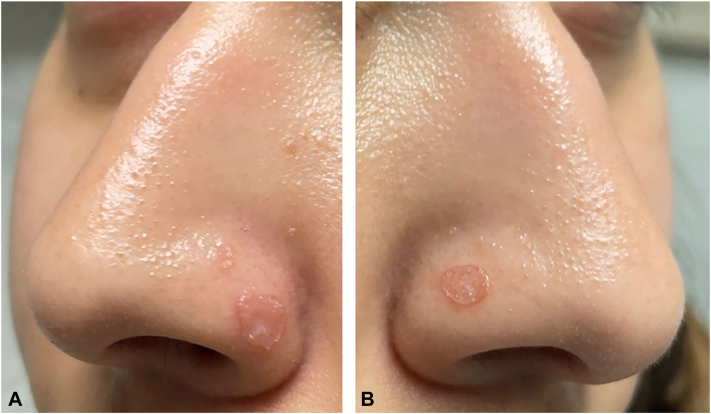
Symmetric papules of nasal alae. **A**, Left. **B**, Right.

A 22-year-old woman presented with a 2-year history of asymptomatic pink papules on the nose that worsened with sun exposure. She denied systemic symptoms and had no significant medical history. The examination was most notable for 2 symmetric, erythematous thin papules with a ridge-like scale on the bilateral nasal alae (Fig 1).

An initial biopsy demonstrated lichenoid dermatitis with mucin deposition, and antinuclear antibody testing was negative. The eruption failed to resolve with topical mupirocin, ketoconazole, topical steroids of various potencies, tacrolimus, and intralesional triamcinolone (Kenalog), prompting referral to a tertiary dermatology center. After evaluation, topical cholesterol/lovastatin cream was added without significant improvement.

**Fig 2:**
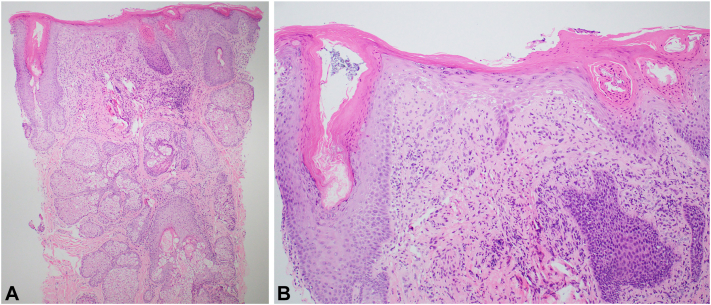
Hematoxylin and eosin-stained sections showing interface dermatitis with follicular plugging in **(A)** low power and **(B)** high power.

**Fig 3:**
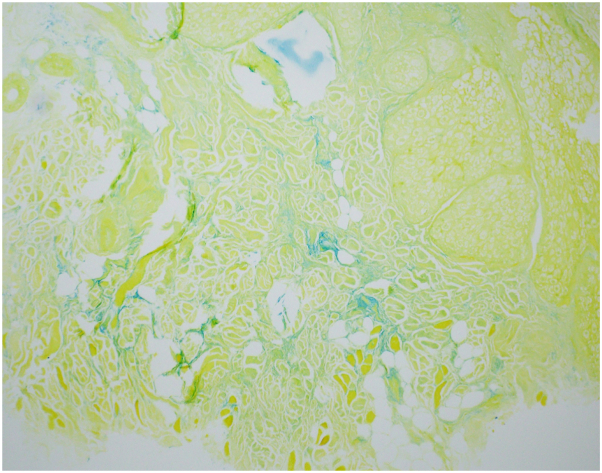
Colloidal iron stain demonstrating increased deep dermal mucin.

A repeat biopsy from the edge of the lesion on the right nasal ala revealed lichenoid interface dermatitis with follicular plugging and superficial- to mid-dermal lymphohistiocytic inflammation (Fig 2). Colloidal iron stain showed abundant deep dermal mucin deposition (Fig 3). Dermoscopy showed persistent keratotic plugs.

**Question 1: What is the most likely diagnosis?**

**A.** Focal actinic porokeratosis
**B.** Lichenoid pseudovesicular papular eruption of the nose
**C.** Actinic keratosis
**D.** Discoid lupus erythematosus (DLE)
**E.** Tinea faciei

**Answer:**

**D.** DLE

## Discussion

The correct diagnosis is DLE. Classic histopathologic features include interface dermatitis with follicular plugging, dermal mucin deposition, and perivascular or periadnexal lymphocytic inflammation, all of which were present in this case.^1^ Clinically, DLE often manifests as erythematous papules or plaques with adherent scale on photo-exposed sites, particularly the face, ears, and scalp.^1^ Negative antinuclear antibody testing does not exclude the diagnosis.

The differential diagnosis includes focal actinic porokeratosis, lichenoid pseudovesicular papular eruption of the nose, actinic keratosis, and tinea faciei.

Actinic porokeratoses have been described as skin-colored lesions with a ridge-like scale on sun-exposed skin, and biopsy demonstrates a pathognomonic cornoid lamella. A few case reports have proposed a distinct subgroup of this condition, known as focal actinic porokeratosis, which features such lesions on the bilateral nasal alae.^2^

Lichenoid pseudovesicular papular eruption of the nose is a rare condition that presents as photosensitive pseudovesicular papules of the nose in young to middle-aged women. Biopsy typically demonstrates a nodular lymphocytic infiltrate with vacuolar basal layer degeneration.^3^

Actinic keratoses are precancerous lesions typically seen in older patients with chronic sun exposure, and biopsy demonstrates atypical keratinocytes without interface change.

Tinea faciei represents a cutaneous infection with dermatophyte fungus, and biopsy typically demonstrates hyphae on periodic acid–Schiff stain.^4^

This case highlights the importance of correlating histopathology, dermoscopy, and clinical findings in diagnosing DLE, particularly when lesions are subtle and symmetric on sun–exposed facial sites. Board-relevant teaching points include recognizing mucin deposition and follicular plugging as key distinguishing features and understanding hydroxychloroquine as first-line systemic therapy. Emerging data show that early initiation of hydroxychloroquine may be beneficial in patients with cutaneous lupus erythematosus, as it could help reduce the risk of progression to systemic disease.^5^

In our case, the patient was counseled extensively on the benefits of starting systemic hydroxychloroquine, but she expressed hesitation about starting oral medication. At present, her treatment regimen includes topical ruxolitinib cream 1.5% and tacrolimus ointment 0.1%, as well as intermittent intralesional Kenalog injections. In addition, she was counseled on strict photoprotection measures given the photosensitive nature of DLE.

## Conflicts of interest

None disclosed.
